# Excerpta

**Published:** 1848

**Authors:** 


### ARTICLE VI.

### EXCERPTA.

A writer in the N. O. Med. Jour., proposes the following remedy in Neuralgic Dysmenorrhœa R. Gum Camphor ʒiiss, Ext. Belladonna ʒss. Sulph. Quinine ʒss---M. Div. in 80 pills.

These pills are to be taken only at each period and at the commencement of pain---Dose two pills every hour until the pain ceases.

Dr. Lockwood of Buffalo, states that he has used *Hornet's nest* as an antispasmodic, and that particularly in hooping cough, he has found it to exert more influence in controlling the paroxysms and shortening the duration of the affection than any other remedy he had ever tried.

Etherization was successfully used in a case of *delirium tremens* reported in the New Jersey Med. Reporter.

The Boston Med. Jour. reports a case of lockjaw cured by chloroform.

The cholera has made its appearance at Berlin.---Reports from St. Petersburg show a decline of the disease there---that it is less severe this year---more easily avoided and cures more numerous. The victims are among the poorer classes who do not abstain from green fruits and vegetables.

Dr. Forbes, who has been claimed by the advocates of Homœopathy as a convert to their doctrines, says, "that the curative powers of nature suffice to explain all the triumphs of homœopathy."

Dr. Allies of Maiagny recommends Ergot of Rye in retention of the urine. He states that it restores the contractile power of the bladder when impaired by over distention, even when all other means have failed; that paralysis of the bladder from cerebral hemorrhage yields to it; and that it is just as efficacious in vesical paralysis, when the lesion of the nervous centres is not clearly established. The dose may be raised to 75 grains a day. M. Trousseau stated in a recent clinical lecture that he had seen great success attend Bretoneau's mode of treating obstinate vomiting during pregnancy. It first occurred to that practicioner, owing to the fact of his patient suffering from violent uterine pain, for the relief of which, believing the vomiting to depend upon its presence, he ordered a belladonna lotion to be applied to the hypogastric region and with the effect of removing both the pain and the vomiting. In subsequent cases the remedy proved as efficacious although no pain was felt---he explained its operation upon the supposition that the vomiting was then sympathetic of irritation of certain of the nerves of the ganglionic system only, produced by enlargement of the uterus.---*Gazette des Hop.*

Dr. Fredericq has observed a line of a red brick color near the free edge of the otherwise normal gums of phthisical patients. The line is very narrow and runs parallel with the edge of the gums, but only opposite the incisors and canine teeth. The researches of the author do not enable him to say whether this sign manifests itself as one of the earliest symptoms of phthisis nor, to declare absolutely that it is seen in no other disease, although he has never yet met with it in such.---*L'Union Medicale.*

